# Amidated Pectin/Nanocellulose Hybrid Cryogel System with a pH-Responsive Release Profile for Small Intestinal Delivery

**DOI:** 10.3390/gels11090700

**Published:** 2025-09-02

**Authors:** Shuhan Feng, Patrick Laurén, Jacopo Zini, Zahra Gounani, Jinfeng Bi, Jianyong Yi, Timo Laaksonen

**Affiliations:** 1 Division of Pharmaceutical Biosciences, Faculty of Pharmacy, University of Helsinki, 00014 Helsinki, Finland; patrick.lauren@helsinki.fi (P.L.); jacopo.zini@helsinki.fi (J.Z.); zahra.gounani@helsinki.fi (Z.G.); 2 Institute of Food Science and Technology, Chinese Academy of Agriculture Sciences (CAAS), Key Laboratory of Agro-Products Processing, Ministry of Agriculture and Rural Affairs, Beijing 100193, China; bjfcaas@126.com (J.B.); yijianyong515@126.com (J.Y.); 3 Faculty of Engineering and Natural Sciences, Tampere University, 33014 Tampere, Finland

**Keywords:** amidated pectin, cellulose nanofibers, cryogel, drug delivery

## Abstract

Cellulose nanofibers and pectin are promising candidates for polysaccharide-based gel carriers. However, their integration into a structurally modified hybrid gel system has not been extensively investigated. In this study, hybrid cryogels with a pH-responsive release profile favoring small intestinal delivery were prepared by freeze-drying various ratios of anionic nanofibrillar cellulose (aNFC) and amidated pectin (AP). Under acidic conditions, carboxylate protonation reduced intermolecular electrostatic repulsion, promoting the formation of the aNFC/AP hybrid gel network. Increasing the AP content enhanced the mechanical strength of the hydrogels and resulted in larger pore sizes after freeze-drying. The hybrid cryogels prolonged the release of a model drug for up to 20–30 min at pH 3.0, while exhibiting rapid release within 1–2 min when the pH exceeded 6.5, due to gel network collapse. The release behavior was governed by both the porous morphology and the crosslinking density of the cryogel scaffolds. These findings demonstrate that aNFC/AP hybrid cryogels possess a well-defined pH-responsive functional window (pH 6.5–7.0) and hold strong potential as oral drug delivery systems targeting the small intestine.

## 1. Introduction

Controlled drug release is a key focus in biomedical technology, aiming to maximize therapeutic efficacy while minimizing non-specific side effects. Over the years, various drug delivery systems (DDS) have been developed to meet these needs. Generally, an ideal DDS should possess several desirable features: biocompatibility, site-directed ability, controlled release, tissue specificity, zero premature release, and high loading capacity, without the potential risk of toxicity in clinical use [1,2]. These properties can often be achieved using natural polymers or macromolecules, which offer high biocompatibility, affordability, availability, renewability, and low toxicity [3]. Combining different materials allows fine-tuning of encapsulation efficiency, bioavailability, and retention time. Such delivery characteristics are influenced by intrinsic polymer properties, including structural modifications, surface charge, and water-swelling capacity, etc. [2]. Chemical modification of polymers enables the formation of hydrogel matrices with controllable porosity, mechanical strength, and ionization behavior, making them promising candidates for the controlled release of drugs [4].

Among natural polymers, cellulose, the most abundant skeletal component of plant cell walls, has been extensively investigated as a material for DDS in biomedical applications due to their excellent biocompatibility, renewable and cost-effective [5,6]. Structurally, cellulose is a syndiotactic polysaccharide composed of β-(1 → 4) linked D-glucopyranose units, forming a dense network of strong intra-/intermolecular hydrogen bonds. This organization promotes self-associate and results in a highly ordered crystalline structure [7,8]. To produce uniform fibril bundles with smaller diameters from this ordered network, intensive mechanical processing methods, often combined with enzymatic pre-treatments, have been employed to obtain nanofibrillar cellulose (NFC) with diameters of 5–40 nm [9]. Compared with native cellulose, NFC exhibits superior properties, including a high elastic modulus (~140 GPa), excellent tensile strength (2–3 GPa), abundant surface functional groups, and a large specific surface area [8,10]. NFC-based hydrogels demonstrate exceptional sustained-release capabilities, largely due to the diffusion barrier created by their three-dimensional nanofiber network and the high density of hydrophilic groups on the NFC surface [4,11]. Furthermore, NFC also could be chemically modified via TEMPO [(2,2,6,6-tetramethylpiperidin-1-yl)oxyl] oxidation to yield anionic nanofibrillar cellulose (aNFC) [12]. Such aNFC has been utilized as a versatile drug delivery matrix for molecules with diverse charge properties and molecular weights [13,14,15].

In addition to cellulose, pectin is another promising polysaccharide for developing DDS and is widely distributed in the primary cell walls of plants. Unlike cellulose, pectin is highly heterogeneous, with its smooth linear region primarily consisting of an α-(1-4)-linked D-galacturonic acid (GalA) backbone, exhibiting varying degrees of esterification and acetylation [16]. Based on the degree of esterification (DM), pectin is classified as high methoxyl pectin (HMP, DM > 50%) or low methoxyl pectin (LMP, DM < 50%), which strongly influences its gelling mechanism and drug loading performance [17]. Numerous pectin-based hydrogels have been explored for drug release applications, including LMP gel system, xyloglucan/pectin gel system, pectin/gelatin gel system, and amidated pectin (AP) gels [18,19,20,21]. Among these, AP has attracted particular interest in drug delivery due to its unique gelling behavior.

Unlike LMP gels, which require divalent cations (e.g., calcium) for gelation, and HMP gels, which require high sugar concentrations (>50% *v*/*v*) in an acidic environment, AP can gel under acidic conditions with only a small amount of calcium [22,23]. Unamidated LMP gels form via the “egg-box” model, in which calcium ions bridge two antiparallel polyuronic acid chains, stabilizing the network through further aggregation. In contrast, AP gelation is not solely dependent on this model but is largely driven by hydrogen bonding between amide groups in the pectin chains [24]. When the pH drops below the pKa (pH 3.5), ionization of the carboxylate groups on the GalA residues is suppressed, reducing electrostatic repulsion between chains [25]. This favors hydrogen bond formation between amide groups, enabling chain aggregation and gelation with minimal calcium. Notably, our recent work demonstrated that AP could form gels in the absence of calcium ions under acidic conditions when the degree of amidation (DA) is sufficiently high [26]. These acid-induced gelling properties motivated us to investigate AP as a pH-responsive material capable of enabling drug release in slightly alkaline environments for DDS. At the same time, the abundant carboxyl groups on the surface of aNFC remain predominantly deprotonated at neutral pH, maintaining dispersion through electrostatic repulsion. This behavior is complementary to that of AP, thereby endowing the AP/aNFC hybrid system with a well-defined pH window for structural disintegration. While AP has been studied as a potential carrier for colon-specific drug delivery, such systems often rely on calcium ion crosslinking [20,27]. To date, no studies have explored the intrinsic pH-responsive gelation behaviors of AP in combination with aNFC for hybrid drug delivery systems.

To date, AP has been explored as a potential drug carrier for colon-specific drug delivery but often requires the assistance of calcium ions [20,27]. In contrast, NFC-based carriers tend to exhibit excessive swelling and uncontrolled drug release, which limits their precision in controlled delivery applications [28]. Therefore, the objective of this study was to develop and characterize calcium-free AP/aNFC hybrid cryogel system for small-intestine-targeted, pH-responsive drug delivery. We hypothesized that the synergistic integration of AP and aNFC, stabilized by glucono-δ-lactone (GDL)-mediated hydrogen bonding and electrostatic interactions, would yield gels that remain stable in gastric acid but undergo rapid disintegration and drug release in the small intestine. Freeze-drying was used as an effective method to remove water from hydrogel systems to produce cryogels, further enhancing their stability, manageability, and facilitating storage [29]. The combination of AP and aNFC is expected to balance the large pore size and poor mechanical strength of AP with the excessive swelling of NFC after freeze-drying, thereby producing a mechanically reinforced scaffold with tunable porosity. Importantly, the system is designed to exhibit a well-defined functional pH window (5.8–7.5), corresponding to the physiological range of the small intestine [30]. Ketoprofen was used as a model drug compound to investigate its release profile from cryogels containing various AP/aNFC ratio in varying pH buffers solutions simulating conditions of the digestive tract environment (i.e., pH 3.0, 5.8, and 7.4). Unlike previously reported AP-Ca^2+^ carriers that target the colon or NFC-only carriers with poor release control, our AP/aNFC hybrid cryogels are designed to achieve gastric protection and rapid release in the small intestine, consistent with gastrointestinal transit times. This innovation highlights their potential as a promising oral drug delivery platform targeting the small intestine, the primary site of nutrient absorption and drug uptake.

## 2. Results and Discussion

### 2.1. Textural Properties of aNFC/AP Hybrid Hydrogels

**Figure 1:**
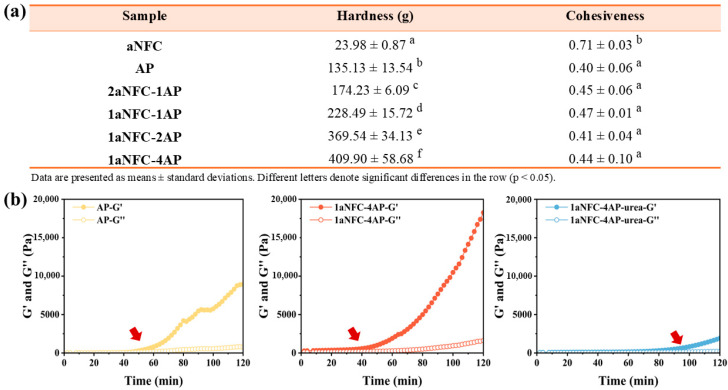
(**a**) Textural characteristics of anionic nanofibrillar cellulose/amidated pectin (aNFC/AP) hybrid hydrogels. Hardness and cohesiveness were determined by texture profile analysis. Values are mean ± SD (*n* = 3). Different letters within the same column indicate statistically significant differences (*p* < 0.05, one-way ANOVA with Duncan’s test). (**b**) Rheological profiles of pure AP gels, aNFC/AP hybrid gels, and urea-treated hybrid gels showing the evolution of storage modulus (G′) and loss modulus (G″) over time. Red arrows indicate the time points where G′ increased.

The textural properties of hydrogels reflect the macroscopic manifestations of polymer crosslinking, with hardness and cohesiveness being the most relevant parameters. Specifically, hardness describes the resistance of the gel network to compressive forces, while cohesiveness refers to its ability to recover structural integrity after deformation [31,32]. The hardness and cohesiveness values of the hybrid hydrogels with varying AP-to-aNFC ratios are summarized in Figure 1a. Pure aNFC hydrogel exhibited the lowest hardness and highest cohesiveness, attributable to their dispersed, flexible network. As reported by Suenaga and Osada [33], TEMPO-mediated oxidation of cellulose microfibrils converts the C-6 hydroxyl groups of glucose units into carboxyl groups, imparting dense surface negative charges. This electrostatic repulsion facilitates microfibril disassembly under mild mechanical shearing, producing aNFC. Although aNFC dispersions can form three-dimensional hydrogels even at low concentrations (0.15–0.4 wt%), these networks rely on physical entanglement rather than covalent or strong physical crosslinking, resulting in relatively weak structures [34]. Consequently, compared to AP hydrogels induced by hydrogen bonding, pure aNFC displays the lowest hardness. Incorporating AP into the aNFC network markedly increased hardness, with higher AP content further enhancing mechanical strength. This may be attributed to the increasing AP content bringing more cross-linking sites, resulting in a higher degree of hydrogen bonding, thereby increasing the mechanical strength of the hydrogel. Furthermore, as the proportion of AP increases, the total amount of polymer in the system also gradually increases, which leads to the distance reduction between the chains, therefore increasing the possibility of having junction zones and enhancing the mechanical properties of the gel network [35]. In terms of cohesiveness, aNFC hydrogels demonstrated greater recovery capacity than AP hydrogels after deformation, reflecting the higher viscosity component of their viscoelastic behavior. In contrast, the higher crosslink density of AP gels conferred greater elasticity under compression. Hybrid hydrogels showed only slightly higher cohesiveness than pure AP gels, indicating that AP predominantly governs the recovery characteristics of the network, regardless of its crosslinking degree.

Rheological analysis further supported the texture results (Figure 1b). For pure AP samples, gelation occurred at ~50 min and the storage modulus (G′) reached ~8800 Pa after 2 h. Upon mixing with aNFC, the gelation onset shifted earlier to ~40 min, and the final G′ increased markedly to ~19,000 Pa, indicating that aNFC not only accelerated the gelation process but also reinforced the mechanical strength of the gel network. This is consistent with the observed increases in hardness and cohesiveness from texture analysis. Notably, when urea was introduced to disrupt hydrogen bonding, the gelation of the hybrid system was significantly delayed to ~90 min, and the final G′ decreased to ~2000 Pa after 2 h. This obvious weakening can be ascribed to the competitive disruption of intermolecular hydrogen bonds by urea, which prevents aNFC chains from forming stable physical crosslinks with the amide groups and hydroxyl groups of AP. These results highlight that hydrogen bonding between aNFC and AP is a major contributor to the physical interactions that govern gelation behavior and mechanical reinforcement.

### 2.2. Structural Analysis of Hybrid aNFC/AP Cryogels

**Figure 2:**
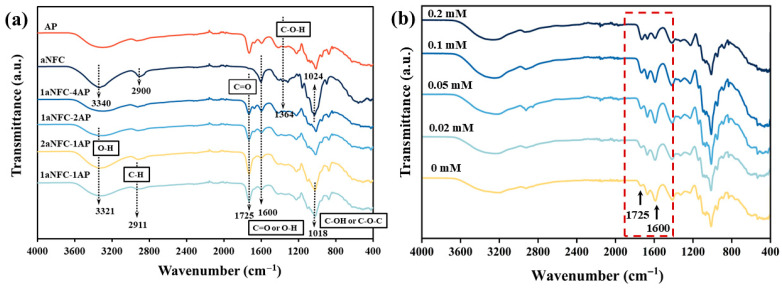
(**a**) FT-IR spectra of pure amidated pectin (AP), pure anionic nanofibrillar cellulose (aNFC), and hybrid aNFC/AP cryogels with different composition ratios. Characteristic absorption changes near 3321, 1725, and 1600 cm^−1^ are indicated with arrows, and the corresponding functional groups are labeled in the text boxes with black borders. (**b**) FT-IR spectra of 1aNFC-4AP cryogels prepared with varying GDL concentrations (0, 0.02, 0.05, 0.1, and 0.2 mM), showing the progressive enhancement of the 1725 cm^−1^ peak and the concomitant decrease of the 1600 cm^−1^ peak as GDL concentration increases, as highlighted in the red frame.

The FT-IR spectra of pure aNFC, pure AP, and hybrid aNFC/AP cryogels are shown in Figure 2. Compared with the characteristic peaks of aNFC, the spectrums of the hybrid cryogels more closely resembled those of AP. In pure aNFC, a broad absorption band at ~3340 cm^−1^, attributed to O–H stretching vibrations from intra- and intermolecular hydrogen bonds, shifted to a lower wavenumber (3321 cm^−1^) upon mixing with AP. This downshift in wavenumber indicates hydrogen bond formation between hydroxyl groups in aNFC and oxygen-containing functional groups in AP, confirming that the interaction involves physical crosslinking [36]. The absorption peaks located at 1725 cm^−1^ and 1600 cm^−1^ correspond to the C=O stretching of ester carbonyl groups and the antisymmetric C=O stretching of carboxylate ions, respectively [37]. Structurally, aNFC, being oxidized cellulose, contains carboxyl groups, whereas the pectin used here was amidated by acetylation modification with NH_4_OH, converting methoxy groups (–COCH_3_) into amide groups (–CONH_2_). Consequently, a distinct peak at 1725 cm^−1^ was observed in pure AP, while pure aNFC exhibited a prominent peak at 1600 cm^−1^.

Notably, as the proportion of aNFC in the hybrid cryogels increased, the intensity of the 1600 cm^−1^ peak decreased, while the 1725 cm^−1^ peak intensified. Theoretically, this trend is unexpected, as increasing AP content should enhance C=O vibrations from ester carbonyl groups (1725 cm^−1^), while C=O vibrations from carboxylate ions (1600 cm^−1^) should remain constant or increase. This abnormal behavior is attributed to changes in the protonation state of aNFC carboxyl groups, influenced by pH fluctuations. Because all cryogels were prepared with the same concentration of glucono-δ-lactone (GDL), the number of hydrogen ions available for protonation was constant. In this basis, cryogels with lower polymer content (i.e., 2aNFC-1AP and 1aNFC-1AP) experienced a higher degree of carboxyl group protonation, producing stronger 1725 cm^−1^ signals [38]. In contrast, samples with higher carboxyl groups density (e.g., 1aNFC-2AP and 1aNFC-4AP) might underwent incomplete protonation, leaving more unprotonated COO^−^ groups and resulting in stronger antisymmetric stretching at 1600 cm^−1^ [39]. To further validate this explanation, we conducted FTIR measurements of 1aNFC-4AP cryogels prepared with varying GDL concentrations (0, 0.02, 0.05, 0.1, and 0.2 mM). The results clearly showed a progressive increase in the 1725 cm^−1^ peak intensity accompanied by a decrease in the 1600 cm^−1^ peak as the GDL concentration increased, confirming that the anomalous peak behavior originates from protonation of carboxyl groups by GDL-generated protons (Figure 2b).

Above result indicates that both hydrogen bonding and electrostatic interactions contribute to AP–aNFC gelation. Gels with a strong 1725 cm^−1^ signal (i.e., high –COOH content) (e.g., 2aNFC-1AP and 1aNFC-1AP) indicate reduced electrostatic repulsion and crosslinking dominated by hydrogen bonding, while 1aNFC-2AP and 1aNFC-4AP, with strong 1600 cm^−1^ signals, indicate a coexistence of electrostatic repulsion and hydrogen bonding due to a higher proportion of unprotonated carboxyl groups.

### 2.3. Morphology of Hybrid aNFC/AP Cryogels

**Figure 3:**
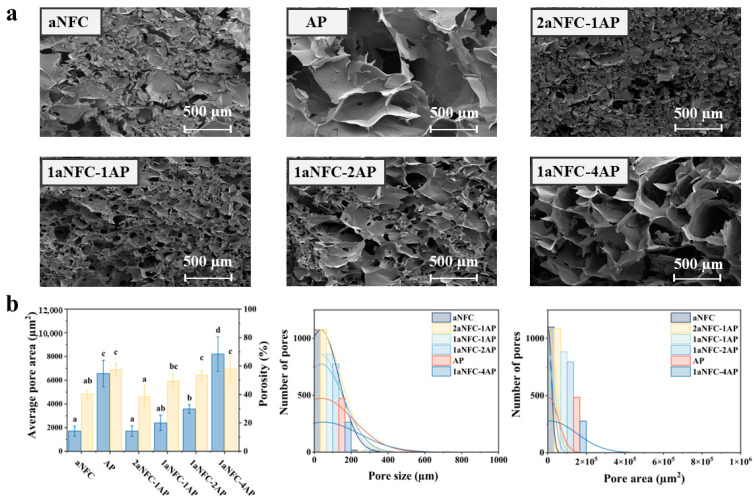
Morphology of hybrid aNFC/AP cryogels. (**a**) Representative SEM micrographs of cryogel cross-sections prepared from aNFC, AP, and hybrid aNFC/AP systems with different composition ratios (2aNFC-1AP, 1aNFC-1AP, 1aNFC-2AP, and 1aNFC-4AP). Scale bars = 500 µm. (**b**) Quantitative pore structure analysis obtained from ImageJ 1.54p: average pore area (blue bars, left Y-axis) and porosity (yellow bars, right Y-axis). Values are mean ± SD (*n* = 3). Different letters indicate statistically significant differences (*p* < 0.05, one-way ANOVA with Duncan’s multiple range test). Middle panel: pore size distribution curves; right panel: pore area distribution histograms for the different cryogel formulations.

The cross-sectional SEM images and pore characteristics of the cryogels are shown in Figure 3. The pure aNFC cryogel exhibited an irregular porous structure with smaller pore sizes, whereas AP-containing hybrid cryogels generally displayed more regular pores with smooth walls and larger diameters. During freeze-drying, as ice crystals grow, aNFC and AP molecules are expelled from the freezing front and concentrated in the non-frozen intergranular regions. Through this cryo-concentration process, polymer chains are forced into closer proximity until ice growth ceases [40]. At the ice crystal boundaries, aNFC and AP segregate and crosslink, forming a continuous network that develops into sheet-like pore walls upon sublimation. Water removal during drying disrupts original hydrogen bonds, which are replaced by new hydrogen bonds between aNFC and AP, as confirmed by FT-IR analysis. The resulting porous architecture is dictated by ice crystal growth behavior, which in turn depends on the hydrophilic groups (–OH, –COOH) present in the hydrocolloid chains under identical freezing conditions. For instance, Xu et al. [41] reported that κ-carrageenan, with abundant carboxyl groups, forms more hydrogen bonds with water, reducing freezable water content. Similarly, partially anionized NFC chains possess numerous hydrophilic carboxyl and hydroxyl groups, enabling stronger interactions with water molecules and more effectively restricting water mobility. In contrast, AP contains amide groups within the HG region and various pentose sugars in the RG-I region—such as arabinose within side chains and xylose in the main chain—which contribute fewer accessible hydrophilic sites compared with aNFC. Consequently, aNFC is more effective in limiting ice crystal growth, leading to smaller ice crystals and reduced pore size. This explains why cryogels with higher aNFC content (e.g., aNFC, 2aNFC–1AP, and 1aNFC-1AP) exhibited smaller pores than those with higher AP content.

As shown in Figure 3b, with increasing AP proportion, the pore size and pore area distributions of the cryogels gradually shifted toward larger scales, and the overall porosity also increased. This trend may be attributed to both the crosslinking strength of the gel network and the steric hindrance it exerts during ice crystal growth. Regarding steric interference, Muhr and Blanshard [42] reported that ice crystals in a gel network grow either by water molecule migration from the network to the ice front or by penetration of the ice front through the micropores of the network. This process requires overcoming both the hydraulic resistance of the gel and the resistance generated by network contraction [43]. Based on the above texture results, the looser, more flexible aNFC network characterized by higher cohesiveness and stretchability may better resist ice front propagation whereas the more rigid AP network, with lower flexibility and cohesiveness, is more easily penetrated by the ice front. With respect to crosslinking strength, previous texture results confirmed that AP content correlates with increased network crosslinking. During freezing, AP–aNFC chains preferentially interact with each other rather than with water molecules, thereby facilitating water mobility and reducing the energy barrier for water migration toward the ice front. This promotes the formation of larger ice crystals, which, after sublimation, yield larger pores. This explains the larger pore sizes and areas observed in 1aNFC-2AP and 1aNFC-4AP. Our previous studies support this mechanism, showing that gels with higher crosslinking density and mechanical strength tend to develop larger porous structures [44].

### 2.4. Swelling of Hybrid aNFC/AP Cryogels Under Difference Acidic Conditions

**Figure 4:**
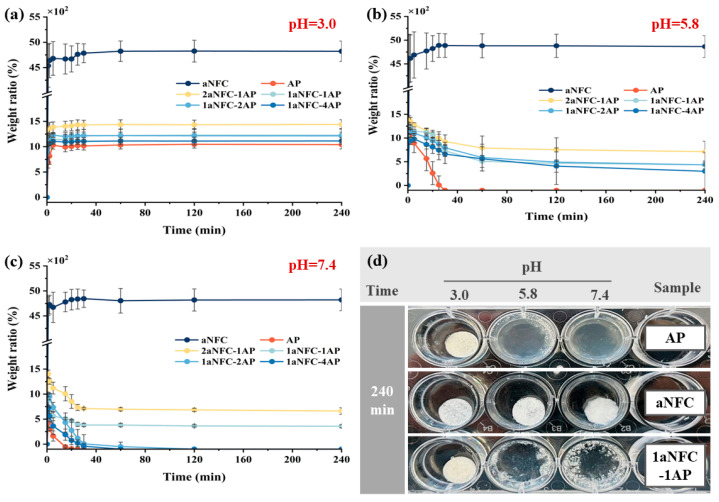
Swelling behavior and macroscopic appearance of cryogels under different pH conditions. (**a**–**c**) Changes in the swelling ratio (weight ratio relative to dry weight, %) of cryogels during immersion in PBS at pH 3.0, 5.8, and 7.4 (37 °C). Data are mean ± SD (*n* = 3); error bars represent SD; (**d**) Representative macroscopic appearances of aNFC, AP, and 1aNFC-1AP cryogels after 240 min of immersion in PBS buffer at different pH values (3.0, 5.8, and 7.4).

The weight ratio of all hybrid aNFC/AP cryogels under 3 different pH conditions (e.g., 3.0, 5.8, and 7.4) is shown in Figure 4. At pH 3.0, simulating the acidic environment of the stomach, all samples exhibited typical swelling behavior. Pure aNFC showed the fastest and greatest weight increase, followed by 2aNFC-1AP and 1aNFC-1AP, while pure AP had the lowest swelling ratio. This rapid weight gain is attributed to the high-water affinity of the freeze-dried porous structure, which creates a steep water gradient between the gel and its environment. The large difference between pure aNFC and other samples can be explained by variations in gel network crosslinking density [45]. Swelling depends on polar interactions between water molecules and hydrophilic groups within the hydrocolloid matrix [46]. As previously noted, aNFC contains more hydrophilic carboxyl than AP, which accounts for its higher swelling capacity. As supported, Ghorbani and Roshangar [47] also found that the increase in hydrophilic groups of hydrocolloids led to a higher swelling ratio of hydrogel scaffolds. In aNFC/AP hybrid cryogels, increasing AP content progressively reduced water uptake, due to the higher proportion of amide groups forming additional hydrogen bonds, which increase hydrogen bonding interaction sites, forming a tough and dense gel network structure with a higher degree of cross-linking, thereby reducing the affinity between the gel network and water molecules [26].

Apart from pure aNFC, all AP-based cryogels exhibited evident degradation under pH 5.8 and pH 7.4 conditions (Figure 4b,c). At pH 5.8, the pure AP sample fully dissolved within 35 min, whereas the hybrid gels reached a degradation plateau after approximately 120 min, leaving behind non-degradable aNFC residues. At pH 7.5, AP cryogels disintegrated completely within 20 min, while the hybrids degraded fully within 40 min. When the pH exceeds the pKa of GalA, deprotonation of carboxyl groups increases electrostatic repulsion between pectin chains, enlarging chain spacing and disrupting hydrogen bonding [26]. This destabilizes the pectin network, leading to the pronounced degradation behavior observed in AP-rich systems. The presence of aNFC slowed this process, indicating that the involvement of aNFC may synergistically interact with AP, suppressing the deprotonation of GalA on the AP molecular chains in a slightly acidic and neutral environment. However, as AP content increased, the resistance of gel network to pH-induced degradation diminished, resembling the behavior of pure AP. Conversely, samples with higher aNFC proportions (e.g., 2aNFC-1AP and 1aNFC-1AP) degraded more slowly and moderately, underscoring the role of aNFC in enhancing structural stability. It should be acknowledged that the swelling ratio cannot fully distinguish between water uptake and matrix degradation, as both processes occur concurrently. Nevertheless, since hydration and degradation are expected to take place simultaneously in the gastrointestinal environment, the swelling data still provide meaningful and physiologically relevant insights into the pH-responsive behavior of the aNFC/AP hybrid cryogels.

### 2.5. Release of the Model Compounds in Hybrid aNFC/AP Cryogels

**Figure 5:**
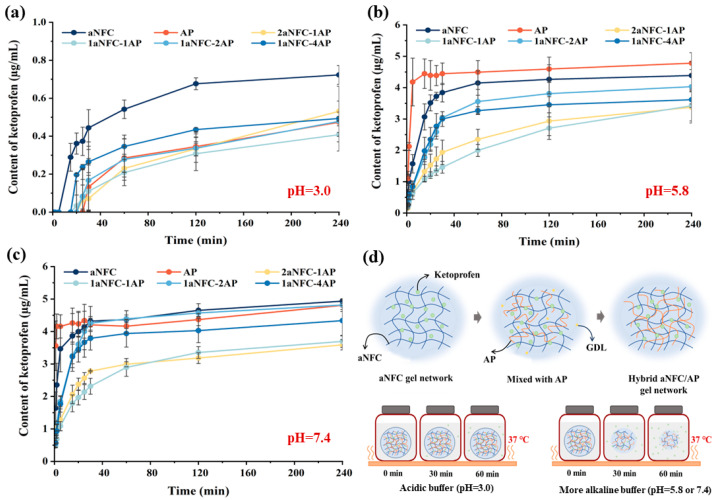
The release of the ketoprofen from pure anionic nanofiber cellulose (aNFC), pure amidated pectin (AP) and all hybrid aNFC/AP cryogels at 37 °C in PBS buffer with pH 3.0 (**a**), 5.8 (**b**), and 7.4 (**c**). Data are mean ± SD (*n* = 3); error bars represent SD; schematic diagram of the formation of aNFC/AP cryogels gel network and release behavior in various pH environments (**d**). GDL represents glucono-δ-lactone; AP and aNFC represents amidated pectin and anionic nanofibrillar cellulose, respectively. The arrow guides the entire process of transforming the system from aNFC gel network to aNFC/AP hybrid gel network.

The ketoprofen release profiles of all cryogel samples at pH 3.0, 5.8, and 7.4 are shown in Figure 5. At pH 3.0, all systems exhibited a lag phase of at least 5 min before gradual drug release began, with final concentrations after 240 min remaining below 0.8 µg/mL (≈16% of the maximum amount). In contrast, at pH ≥ 5.8, ketoprofen release commenced within 1–2 min and reached up to 5 µg/mL (≈100%). Under acidic conditions, protonation of carboxylate groups suppresses electrostatic repulsion between polymer chains, promoting aggregation of aNFC and AP. Consequently, ketoprofen becomes more tightly retained within the pore walls formed via non-covalent crosslinking during cryo-concentration, making release more difficult. Upon transfer to higher pH, deprotonation of AP and aNFC increases electrostatic repulsion, destabilizing the network and accelerating its disintegration, thereby facilitating rapid and complete drug release. Thus, compared to acidic conditions, ketoprofen is released more efficiently at neutral to mildly acidic pH due to gel network breakdown.

Cryogels with different aNFC/AP ratios showed significantly different release behaviors in acidic, slightly acidic, and neutral environments. At pH 3.0, pure aNFC delayed ketoprofen release by only ~5 min, whereas AP-containing cryogels delayed release by at least 20 min. This difference may be related to the higher swelling capacity and weaker crosslinking of aNFC compared with AP. Drug release from cryogels generally involves two stages: (i) a rapid swelling phase dominated by capillary water absorption and hydrocolloid rehydration, followed by (ii) a slower solute leaching phase [48]. Upon immersion, interconnected pores fill quickly via capillary action, with water acting as a “mobile solute.” Subsequently, water, as an “immobile solute”, diffusion through the solid polymer phase is much slower [49]. The weaker crosslinked network of aNFC facilitates faster water transport in the solid phase, accelerating drug leaching. In contrast, AP introduces non-covalent crosslinking with aNFC, producing a denser network that traps ketoprofen more effectively and slows rehydration. Banerjee et al. [35] similarly observed slower rehydration in denser, more entangled gels. Interestingly, during the first 120 min, cryogels with higher crosslinking (e.g., 1aNFC-4AP and 1aNFC-3AP) released ketoprofen faster than moderately crosslinked samples (e.g., 1aNFC-1AP and 2aNFC-1AP), suggesting that release rate is influenced not only by crosslinking density but also by pore size. The smaller, denser pores of 1aNFC-1AP and 2aNFC-1AP may hinder capillary penetration. Once pores are saturated under capillary forces, further water uptake is governed by the diffusion of trapped air after the waterfront reaches the top of the pores [50]. Hence, smaller, denser pore structures may cause greater air entrapment, slowing ketoprofen leaching.

At pH 5.8 and 7.4, the pure AP cryogel exhibited the fastest ketoprofen release, followed by aNFC, 1aNFC-2AP, and 1aNFC-4AP, whereas 2aNFC-1AP and 1aNFC-1AP released the drug most slowly. In these higher-pH environments, drug release from AP gels was faster than from aNFC gels, indicating that AP is more susceptible to pH-induced degradation due to its more efficient deprotonation compared with aNFC. Combined with the water swelling data (Figure 4), the release profiles paralleled the degradation behaviors, supporting our conclusion that drug leaching occurs primarily via gel network collapse. As under acidic conditions, the macroporous structures of 1aNFC-2AP and 1aNFC-4AP facilitated rapid and thorough buffer penetration into the pore walls, leading to unrestricted network disintegration and accelerated drug release. In contrast, the smaller pores of 1aNFC-1AP and 2aNFC-1AP likely restricted buffer penetration due to increased air entrapment, thereby slowing gel collapse and delaying drug release.

As illustrated in Figure 5d, the formation process of aNFC/AP cryogels determines their highly pH-sensitivity. Protonation of GalA residues on AP chains was achieved via hydrogen ions generated by the slow hydrolysis of GDL, thereby eliminating electrostatic repulsion between pectin chains. This enabled the formation of an AP gel network through hydrogen bonding between amide groups, which intersected with the pre-existing, weakly crosslinked aNFC network. During freeze-drying, cryo-concentration promoted additional non-covalent interactions between the AP and aNFC networks, producing the pore walls of the cryogel scaffold after ice sublimation. This structure remained stable under acidic conditions, delaying drug release for up to 20–30 min. In contrast, under slightly acidic or neutral conditions, rapid deprotonation destabilized the network, leading to scaffold collapse and high drug release within 20 min. A study of fluorescent capsules reported that the median gastric transit time was 22 min, while the median small intestinal transit time was 198.5 min (IQR 157–240.5 min) [51]. This suggests that aNFC/AP cryogels can generally protect acid-sensitive drugs in the gastric environment while achieving rapid release in the healthy small intestine, typically at a pH of 6.5–7.5 [52]. Given that aNFC/AP cryogels exhibit significant release within 20 min at a pH greater than 5.8, it is reasonable to infer that under certain pathological or atypical physiological conditions, when the small intestinal pH exceeds the upper physiological limit (e.g., approaching 8.0), the cryogel network would undergo even faster disintegration and drug release. However, such conditions are relatively uncommon in healthy adults.

### 2.6. Non-Linear Release Data Fitting Using Korsmeyer-Peppas and Weibull Model

**Figure 6:**
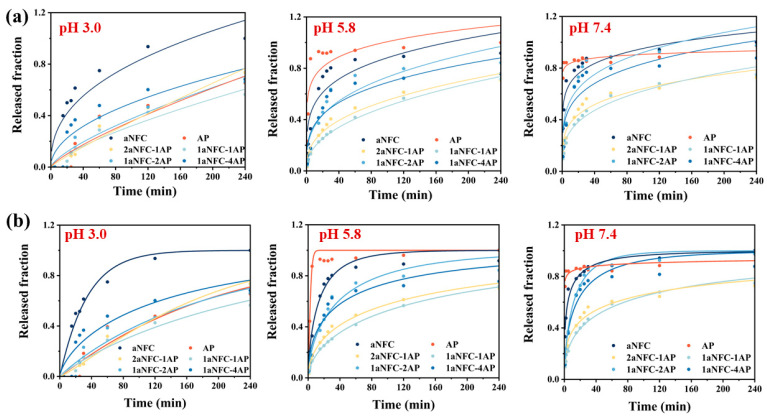
Release kinetics of ketoprofen from pure anionic nanofibrillar cellulose (aNFC), pure amidated pectin (AP), and hybrid aNFC/AP cryogels at 37 °C in PBS buffer with pH 3.0, 5.8, and 7.4. (**a**) Fitting of the experimental release data (symbols) with the Korsmeyer–Peppas model (solid lines). (**b**) Fitting of the same data with the Weibull model.

Korsmeyer-Peppas (K-P) analysis was used to analyze the different release mechanisms under different pH conditions, as shown in Figure 6a and Table 1. At pH 3.0, aNFC exhibited *n* ≈ 0.40, close to the Fickian diffusion threshold, and a relatively high *k*, indicating a diffusion-driven and relatively fast release [53]. By contrast, AP showed a much higher *n* (*n* ≈ 0.78), suggesting anomalous transport involving polymer relaxation/swelling, but its *k* was very low, reflecting suppressed initial release. The aNFC/AP hybrids displayed intermediate *n* values (0.49–0.88) and reduced *k*, suggesting that incorporation of aNFC promoted more controlled diffusion. At pH 5.8, both AP and aNFC showed very low *n* (<0.25), indicative of burst-like surface diffusion. The hybrids, however, exhibited moderately higher *n* (0.28–0.43), together with lower *k*, pointing to the contribution of the hybrid network in slowing down the initial release and shifting the mechanism closer to Fickian diffusion. At pH 7.4, AP nearly lost release control (*n* = 0.03), whereas hybrids retained higher *n* values (0.22–0.30) and lower *k*, suggesting that the presence of aNFC provided additional hydrogen-bonding and steric constraints, thereby reducing burst release and enabling more diffusion-governed release behavior [54]. These findings are consistent with the rheological and textural evidence that aNFC/AP hybrids form stronger, more crosslinked networks, particularly under neutral conditions, which mitigate premature release.

However, the fitting quality of the K-P model was modest in some cases (e.g., *R^2^* as low as 0.63–0.83), reflecting the fact that K-P is most suitable for the initial 60% release and does not fully capture later-stage erosion or matrix collapse. To further elucidate the mechanism of drug release from the aNFC/AP cryogels, the release profiles were fitted to the empirical Weibull model, which has been widely employed to describe controlled release systems and discriminate between diffusion- and erosion-governed processes [55]. As displayed in Table 1, the model provided consistently high goodness-of-fit (*R^2^* = 0.87–0.99 for most samples), outperforming the K-P model. At pH 3.0, pure aNFC exhibited a small *λ* value (34.5) and a *β* below unity (0.41), indicating a rapid and diffusion-dominated release. In contrast, AP showed a much larger *λ* (195.1) and *β* close to unity (1.04), corresponding to slower, near first-order kinetics [56]. This behavior is consistent with the rheological observation that AP undergoes acid-induced gelation, forming a transiently stronger network that retards diffusion. The aNFC/AP hybrids showed intermediate *λ* values (142.5–196.8) and *β* values ranging from 0.70 to >1.0. Notably, the 2aNFC-1AP formulation displayed *β* > 1, suggesting a sigmoidal profile associated with polymer relaxation and delayed network opening. These results imply that while aNFC alone cannot prevent burst release under acidic conditions, the incorporation of AP provides additional hydrogen bonding and steric hindrance, resulting in slower and more regulated diffusion. At pH 5.8, both aNFC (*λ* = 15.4, *β* = 0.69) and AP (*λ* = 2.92, *β* = 1.30) released rapidly, with AP showing almost immediate release due to loss of acid-induced structural stability. In contrast, the hybrids exhibited much larger *λ* values (43.5–164.7) and *β* < 1 (0.49–0.63), corresponding to slower overall release. At pH 7.4, AP essentially lost its release control, with *λ* approaching zero (0.025) and *β* ≈ 0.10, indicative of an almost instantaneous burst release. In comparison, hybrids maintained large *λ* values (82.1–142.5) and moderate *β* values (0.36–0.68), showing sustained and diffusion-driven release. Particularly, 1aNFC-4AP exhibited the highest *λ* (142.5), aligning with its superior mechanical hardness and higher G′ observed in rheological tests. These results confirm that the hybrid network retains structural integrity under neutral conditions, where AP alone is incapable of resisting matrix collapse.

Although the important influence of pore size and porosity on release rate has been mentioned above, it is clear that drug release from hybrid cryogel is governed by multiple concurrent. In addition to diffusive transport through aqueous channels, the release is influenced by gel network collapse, polymer relaxation, water swelling, and the disruption of hydrogen bonding. These interconnected processes diminish the direct predictability of release kinetics based solely on pore size. However, the general trend remains consistent: samples with smaller effective pore sizes and stronger gel mechanisms networks (as shown by rheology and texture analysis) exhibited slower initial release and higher λ values in Weibull modeling, whereas larger pores facilitated faster release. This highlights that pore size is a necessary but not sufficient descriptor of release and must be considered in conjunction with the dynamic structural evolution of the cryogel matrix. Overall, these findings support the conclusion that aNFC/AP hybrid cryogels suppress burst release and maintain diffusion-controlled release across gastrointestinal pH conditions, providing a robust platform for small-intestinal drug delivery. This system is particularly promising for acid-labile small molecules such as proton pump inhibitors (e.g., omeprazole) to ensure release and absorption in the high pH environment of the small intestine [57]. In addition, such systems are also particularly relevant for gastrically irritating drugs (e.g., ketoprofen, ibuprofen, diclofenac), which can cause gastric mucosal injury if released in the stomach, and for acid-sensitive biotherapeutics (e.g., peptides and proteins such as insulin, calcitonin, and GLP-1 analogs), which undergo rapid degradation in gastric acid and pepsin [58,59]. By protecting the payload in the stomach and ensuring rapid release in the small intestine (pH 6.0–7.5), the cryogels meet clinically relevant requirements for these therapeutic classes.

## 3. Conclusions

In this study, we developed an aNFC/AP cryogel DDS with a pH-responsive release profile favoring neutral or slightly alkaline conditions. As an acidic polymer, AP formed stable acid-induced gel networks below pH 3.0 through the combined effects of carboxyl protonation and hydrogen bonding between amide groups. This AP network intermingled with the pre-existing aNFC network, and under cryo-concentration during freezing, additional crosslinking occurred. After ice sublimation, these interactions produced the pore wall structures of the cryogel scaffold. Increasing AP content enhanced mechanical strength and crosslinking density, which in turn influenced the scaffold morphology with higher crosslinking strength yielded larger pores.

The aNFC/AP hybrid cryogel system effectively inhibited model drug release under acidic conditions (pH < 3.0) for up to 20–30 min and rapidly released the drug within 1–2 min under slightly acidic to neutral conditions (pH > 5.8) via structural disintegration of the gel network. This combination of excellent acid resistance and alkaline degradability highlights its potential as an oral DDS targeting drug release in the small intestine. The hybrid system mitigates the excessive swelling seen in pure aNFC and the large pore sizes of pure AP, offering acid-induced low electrostatic repulsion and structural stability that limit drug release in the gastric environment to less than 16%. In contrast, under small intestinal conditions (pH 6–8), the hybrid cryogels completed drug release within approximately 120 min. In addition, kinetic modeling provided further mechanistic insights, which confirmed that the hybrids suppressed burst release and maintained diffusion-controlled kinetics across gastrointestinal pH conditions.

These findings demonstrate that aNFC/AP hybrid cryogels provide gastric protection and enable rapid, diffusion-controlled release in the small intestine. The system is particularly relevant for acid-labile drugs (e.g., omeprazole), NSAIDs prone to gastric irritation (e.g., ketoprofen), and acid-sensitive peptides/proteins, highlighting its potential as a pH-responsive platform for oral intestinal delivery. Nevertheless, this study has certain limitations. The release behavior was evaluated under gastric (pH 3.0) and small-intestinal (pH 5.8 and 7.4) conditions, which represent the physiologically relevant environments for oral absorption. The performance of the cryogels at pH ≥ 8.0 (occasionally encountered in the colon or under pathological states) was not investigated in this work. Given that our data at pH 7.4 already demonstrated rapid network disintegration and accelerated release, it is reasonable to expect even faster release at higher pH; however, future studies will be required to directly confirm the stability and release profiles under such alkaline conditions. Overall, this research reveals the robustness of this platform as a calcium-free, pH-responsive oral drug delivery system with strong translational potential.

## 4. Materials and Methods

### 4.1. Materials

An aNFC hydrogel (Fibdex™, lot 12126; 2.27% *w*/*w*) was purchased from UPM-Kymmene Oyj (Helsinki, Finland). High methoxyl pectin (HMP) derived from apples (Classic APA170; DM = 75 ± 3%; ash < 1%) was kindly provided in dry powder form by DSM Andre Pectin (Shandong Andre Group Co., Ltd., Yantai, China). Pectin was purified with 80% ethanol to remove soluble low-molecular-weight sugars, followed by amidation as described in our previous work [26]. Briefly, purified pectin was dissolved in deionized water and reacted with 60% isopropyl alcohol containing 4 mol/L NH_4_OH at 4 °C for 40 min. The reaction mixture was then rapidly washed with 60% isopropyl alcohol, and the pH was adjusted to 4.0 ± 0.3 using 60% isopropyl alcohol containing 1 mol/L HCl to terminate the reaction. The product was washed three times with 60% isopropyl alcohol to remove residual ammonia and chloride, followed by washing with pure isopropyl alcohol and drying overnight at 40 °C. The amidated pectin obtained had a DM of 40.77 ± 0.06%, a DA of 49.76 ± 2.76%, and a molecular weight of 2.29 × 10^5^ [26].

GDL, ketoprofen (BCCH4038, ≥98%), potassium dihydrogen phosphate, and dipotassium phosphate were purchased from Sigma-Aldrich (Sigma-Aldrich Finland Oy, Espoo, Finland). Hydrochloric acid (HCl), sodium hydroxide (NaOH), ammonium hydroxide (NH_4_OH), isopropanol, and urea were obtained from Shanghai Aladdin Biochemical Technology Co., Ltd., (Shanghai, China). All buffer solutions were prepared using double-distilled ultrapure water.

### 4.2. Preparation of the aNFC/AP Hybrid Cryogels

aNFC/AP hybrid cryogels were prepared via slow acid induction through GDL hydrolysis. In aqueous solution, GDL gradually hydrolyzes to gluconic acid, providing a gentler and slower pH reduction than conventional acidulants. Amidated pectin powder was first dissolved in deionized water at room temperature overnight to prepare stock solutions at concentrations of 1.0, 2.0, 4.0, and 8.0% (*w*/*v*). Each pectin stock solution was mixed with aNFC hydrogel using two 10 mL syringes connected by a short rubber tube. Specifically, 2.39 mg of ketoprofen and 0.4 M GDL were dissolved in 3 mL of AP stock solution, while the other syringe was filled with 3 mL of aNFC hydrogel diluted to 2.0% (*w*/*v*) fiber content with deionized water. The syringes were alternately pressed for 4 min to produce a homogeneous dispersion, which was then transferred into a 48-well plate and left at room temperature for 12 h to complete gelation. Each well contained a 1 mL precursor solution to ensure uniform sample height. The final hybrid hydrogels contained 0.2 M GDL, 1.0% aNFC, and varying pectin concentrations (0.5%, 1%, 2%, and 4%). The total ketoprofen incorporated in the precursor solution was 2.39 mg, corresponding to ~0.40 mg per cryogel (1 mL wet weight), assuming quantitative entrapment during gelation. These were designated as 2aNFC–1P, 1aNFC–1P, 1aNFC–2P, and 1aNFC–4P, corresponding to aNFC/AP ratios of 2:1, 1:1, 1:2, and 1:4, respectively. Pure aNFC and pure AP hydrogels (2.0% *w*/*v*) were prepared as controls.

After freezing overnight at −80 °C, all aNFC/AP hydrogels were transferred to a vacuum freeze dryer (CoolSafe Touch 1110-4, LaboGene A/S, Allerød, Denmark). Lyophilization was carried out at a chamber pressure below 1 Pa with the cold trap maintained at −110 °C. The resulting cryogels were stored in a desiccator until further analysis.

### 4.3. Textural Analysis of aNFC/AP Hybrid Hydrogels

The textural properties of aNFC/AP hybrid hydrogels were evaluated using texture profile analysis (TPA) with a texture analyzer (Stable Micro Systems Ltd., Godalming, Surrey, UK) equipped with a radiused cylinder probe (P/0.5). Tests were performed at room temperature at a constant speed of 1 mm/s until the sample height was compressed to 40% of its original value. The trigger force was set to 5 g with automatic triggering. Each measurement was repeated at least five times.

### 4.4. Swelling and Degradation Behavior of aNFC/AP Hybrid Cryogels

The swelling and degradation behaviors of aNFC/AP hybrid cryogels under different pH conditions were assessed using a gravimetric method adapted from our previous work [26]. Briefly, cryogels were immersed in PBS solutions at pH 3.0, 5.8, or 7.4 and incubated at 37 °C. At predetermined time intervals (1, 2, 5, 10, 15, 20, 25, 30, 60, 240, and 480 min), samples were removed, gently blotted with filter paper to remove surface water, and weighed. All measurements were performed in triplicate. The weight ratio was calculated according to Equation (1)

(1) $$\mathrm{WR}=\left[\frac{w_{t}-w_{0}}{w_{0}}\right]\times 100\;$$

where WR is the weight ratio of aNFC/AP hybrid cryogel (%), *W_t_* is the weight of the soaked cryogel at time *t*, *W_0_* is the weight of the original aNFC/AP hybrid cryogel.

### 4.5. In Vitro Drug Release Studies

Equal volumes of aNFC/AP hybrid cryogels were placed into 40-mesh steel baskets, with the non-mesh side sealed using Parafilm. The sealed baskets were submerged in glass bottles containing 80 mL of PBS at pH 3.0, 5.8, or 7.4. The bottles were incubated in a 37 °C shaker (Innova 4400, ALLERGAN Inc. Irvine, CA, USA) at 300 rpm for 4 h. At predetermined time points (1, 2, 5, 10, 15, 20, 25, 30, 60, 240, and 480 min), 200 µL samples were withdrawn and replaced with an equal volume of fresh buffer. All experiments were performed in triplicate.

### 4.6. Quantification of Released Model Compounds

Ketoprofen concentrations in the release samples were quantified using ultra-performance liquid chromatography (UPLC; Waters Corporation, Milford, Massachusetts, USA). Aliquots (2 μL) were injected onto a Luna Omega Polar C18 column (50 × 2.1 mm i.d., 1.6 μm particle size) and eluted with a mobile phase of acetonitrile and 15 mM phosphate buffer (pH 2.0) at a 60:40 (*v*/*v*) ratio and a flow rate of 0.5 mL/min. Detection was performed by UV absorbance at 255 nm, with a ketoprofen retention time of 1.01 min. External standards ranging from 0.01 to 10 µg/L were used for qualitative and quantitative analysis.

### 4.7. Fourier Transform Infrared (FT-IR) Spectroscopy

FT-IR spectra of aNFC/AP hybrid cryogels were recorded using a Nicolet™ iS50 FTIR spectrometer (Thermo Fisher Scientific Inc., Waltham, MA, USA) equipped with an iS50 ATR multirange sampling station. Spectra were collected over the wavenumber range of 400–4000 cm^−1^.

### 4.8. Scanning Electron Microscopy (SEM) Measurements

Scanning electron microscopy (SEM; Quanta 250, FEI Company, Eindhoven, The Netherlands) was used to analyze the porous morphology of freeze-dried aNFC/AP hybrid cryogels. Samples were cut with a knife to expose cross-sections and sputter-coated with gold for 30 s (Agar Scientific, Stansted, UK) prior to imaging.

### 4.9. Scanning Electron Microscopy (SEM) Measurements

The pore morphology of cryogels was analyzed from cross-sectional SEM images using ImageJ (version 1.54p, National Institutes of Health, Bethesda, MD, USA). Images were calibrated with the scale bar, converted to grayscale, and binarized by threshold adjustment to distinguish pores from the matrix. Porosity was calculated as the percentage of pore area relative to the total image area, while pore size distribution was obtained using the “Analyze Particles” function. At least three images were analyzed per sample, and results are reported as mean ± SD.

### 4.10. Release Kinetics Modeling

To investigate the drug release mechanisms, the cumulative release data (*Mt*/M∞ vs. time) were fitted to the Korsmeyer-Peppas (K-P) and Weibull models using the non-linear curve fitting (NLFit) module in OriginPro 2024. Data were first normalized by dividing the cumulative drug release at each time point (*Mt*) by the maximum release value (*M*∞), yielding a dimensionless release fraction (0–1).

For the Korsmeyer–Peppas model, the semi-empirical equation:

$$M_{t}/M_{\infty }=kt^{n}$$

was applied, where *k* is the release rate constant incorporating structural and geometric characteristics of the matrix, and *n* is the release exponent indicative of the transport mechanism. The kinetic constant *k* was constrained to positive values, and the release exponent *n* was restricted to the range 0–1, as recommended for thin polymer matrices [60].

The Weibull model was expressed as:

MtM∞=1−exp−tλβπr2

where *λ* is the scale parameter, inversely related to the overall release rate, and *β* is the shape parameter describing the release profile. The full release profile (0–100%) was used for fitting. (*β* < 1: diffusion-like; *β* = 1: first-order kinetics; *β* > 1: sigmoidal release suggesting relaxation/erosion involvement) [56]. Both parameters were constrained to positive values, and the entire release dataset was used for fitting.

Goodness-of-fit was evaluated using the coefficient of determination (*R*^2^, COD in Origin). For multi-parameter models such as Weibull, the adjusted *R*^2^ (Adj. *R*^2^) was additionally inspected to avoid overestimation due to model complexity.

### 4.11. Statistical Analysis

Unless otherwise stated, all experiments were performed in triplicate, and results are reported as mean ± standard deviation (SD). Statistical analysis was conducted using OriginPro 2024 and SPSS 16.0. Data were first tested for normality using the Shapiro–Wilk test and for homogeneity of variance using Levene’s test. When the assumptions of normal distribution and homogeneity of variance were satisfied, one-way analysis of variance (ANOVA) followed by Duncan’s multiple range test was applied to determine significant differences among replicates. In cases where the assumption of homogeneity of variance was violated, Tamhane’s T2 test was used instead as a more robust post hoc analysis. Statistical significance was set at *p* < 0.05.

## Figures and Tables

**Table 1:** Fitted kinetic parameters of ketoprofen release from all cryogels under different pH conditions.

pH	Sample	Korsmeyer–Peppas			Weibull		
		*k*	*n*	*r* ^2^	*λ*	*β*	*r* ^2^
3.0	aNFC	0.12	0.404	0.857	34.48	0.413	0.97
	AP	0.009	0.782	0.865	195.057	1.036	0.9
	2aNFC–1AP	0.006	0.884	0.951	179.341	1.192	0.97
	1aNFC–1AP	0.011	0.728	0.934	260.828	0.91	0.95
	1aNFC–2AP	0.013	0.716	0.891	196.779	0.942	0.92
	1aNFC–4AP	0.051	0.493	0.831	142.517	0.704	0.87
5.8	aNFC	0.308	0.228	0.776	15.443	0.685	0.97
	AP	0.538	0.136	0.572	2.922	1.3	0.95
	2aNFC–1AP	0.101	0.368	0.953	133.777	0.521	0.98
	1aNFC–1AP	0.069	0.431	0.993	164.65	0.584	0.99
	1aNFC–2AP	0.166	0.322	0.861	43.509	0.634	0.95
	1aNFC–4AP	0.189	0.281	0.814	53.712	0.495	0.9
7.4	aNFC	0.493	0.143	0.83	5.524	0.413	0.96
	AP	0.774	0.034	0.63	0.025	0.102	0.63
	2aNFC–1AP	0.218	0.234	0.91	82.061	0.36	0.95
	1aNFC–1AP	0.155	0.302	0.96	91.66	0.455	0.99
	1aNFC–2AP	0.329	0.224	0.81	93.33	0.675	0.99
	1aNFC–4AP	0.301	0.221	0.76	142.517	0.56	0.92

Note: Parameters *k* and *n* correspond to the Korsmeyer–Peppas model, where *k* is the kinetic constant and *n* the release exponent. Parameters *λ* (scale factor) and *β* (shape factor) correspond to the Weibull model. *R*^2^ values represent the coefficient of determination (COD, Origin output), reflecting the goodness of fit.

## Data Availability

The original contributions presented in the study are included in the article; further inquiries can be directed to the corresponding authors.

## Abbreviations

The following abbreviations are used in this manuscript:

aNFC	Anionic Nanofibrillar Cellulose
AP	Amidated Pectin
DDS	Drug Delivery Systems
NFC	Nanofibrillar Cellulose
GalA	D-Galacturonic Acid
HMP	High Methoxyl Pectin
LMP	Low Methoxyl Pectin
DA	Degree of Amidation
GDL	Glucono-δ-Lactone
